# Case Report: Hemorrhagic–Thrombotic Escalation After Intraprocedural Rupture During Stent-Assisted Coiling: A Case-Based Narrative Review and Staged Communication Model

**DOI:** 10.3390/jcm15114056

**Published:** 2026-05-24

**Authors:** Kosei Goto, Nobuo Kutsuna, Takuto Nishihara, Kotaro Makita

**Affiliations:** 1 Department of Neurosurgery, Fukujukai Adachi Tobu Hospital, Tokyo 121-0816, Japan; k.goto@fukujukaigr.or.jp (K.G.);; 2 Department of Stress and Invasiveness Control, Toho University School of Medicine, Tokyo 153-8515, Japan

**Keywords:** anterior communicating artery aneurysm, intraprocedural rupture, stent-assisted coiling, acute in-stent thrombosis, external ventricular drainage, team communication, case report, staged communication model

## Abstract

Intraprocedural rupture (IPR) during stent-assisted coiling (SAC) after stent deployment can create a narrow and rapidly changing management problem: hemorrhage control, anticoagulation reversal, acute thrombotic occlusion, and postprocedural cerebrospinal fluid diversion may all become urgent within the same clinical sequence. We report a fatal IPR during SAC of an unruptured anterior communicating artery (AComA) aneurysm and use the case as an anchor for a targeted case-based narrative review. A 71-year-old woman underwent SAC for a 5.1-mm posteriorly directed AComA aneurysm with a bleb after treatment for vertebrobasilar ischemia. Fourth-coil insertion produced tactile resistance and contrast extravasation. Protamine reversal and temporary A1 flow control reduced the leak, but filling defects then developed from the internal carotid artery terminus to the A1 and M1 segments, requiring rescue thrombectomy. Computed tomography showed subarachnoid hemorrhage and intraventricular hemorrhage; same-day progression with hydrocephalus required bilateral external ventricular drainage. The patient died on postoperative day 7. This case highlights IPR during SAC as a time-dependent hemorrhagic–thrombotic escalation rather than a single technical event. We propose a staged assistant–operator communication model for risk mapping, rupture recognition, hemostatic-route preservation, thrombotic surveillance, and transition to computed tomography, external ventricular drainage, and intensive care.

## 1. Introduction

Endovascular treatment of an unruptured intracranial aneurysm (UIA) is performed in a setting where the accepted margin for procedural harm is narrow. Complication prevention is therefore not only a matter of device handling; it also depends on indication selection, team communication, and the ability to recover from an unexpected event [1]. For trainees and junior operators, education must go beyond routine catheter and coil manipulation and include how to think, speak, and prepare when a complication begins [2].

Existing reports provide useful but separate pieces of this problem. Checklists for cerebral aneurysm embolization and aneurysm perforation describe immediate tasks such as confirmation of rupture, blood pressure control, anticoagulation reversal decisions, coil packing, and team role allocation [3,4]. A management review summarizes perforation and thromboembolic rescue during endovascular aneurysm treatment [5], and a neurointerventional procedural checklist has been associated with better staff communication and fewer total adverse events [6]. These reports define important steps, but they do not fully specify what a junior assistant should watch in real time, which observation should be verbalized first, and what should be prepared next.

During SAC, the problem can change from hemorrhage to thrombosis within minutes. In a multicenter study of IPR during coiling of UIAs, Aoki and colleagues identified blebs, a small neck, and AComA or posterior communicating artery location as independent risk factors; rapid flow control using a balloon-guiding catheter was associated with better outcomes after IPR [7]. Nii and colleagues reported acute in-stent thrombosis in 11 of 185 SAC procedures for UIAs, with a mean onset of 19 min after stent placement [8]. AComA-specific SAC series also include thromboembolism and procedural leakage among recognized complications [9,10]. A meta-analysis of endovascular treatment for AComA aneurysms estimated pooled rates of intraprocedural complications (9.6%), thrombosis (6.1%), rupture (4.2%), and coil prolapse (4.7%) [11]. Together with the reported association between heparin reversal and ischemic lesions after IPR, these data support viewing SAC-associated rupture as a dynamic hemorrhagic–thrombotic event rather than as an isolated perforation [12].

This case report uses an illustrative fatal case of IPR during SAC of an unruptured AComA aneurysm to synthesize the relevant literature from the viewpoint of a junior assisting operator. The purpose is not to introduce a new bailout technique or to claim that IPR itself is unique. Rather, the aim is to organize the hemorrhagic, thrombotic, and postprocedural phases into a time-dependent communication model for preparation, intraoperative prompting, and handoff.

## 2. Case-Based Narrative Review Approach

This case report is supported by a targeted case-based narrative review rather than a systematic review or scoping review. The illustrative case was used to define the clinical sequence, and the literature search was then mapped to each phase of that sequence. On 31 March 2026, PubMed was searched using title/abstract text-word blocks for seven concepts: IPR, aneurysm perforation, or extravasation combined with coiling or SAC; AComA aneurysm combined with rupture, perforation, or extravasation; acute, intraprocedural, or periprocedural thrombosis or thromboembolism after SAC; rerupture, IVH, or hydrocephalus after IPR; EVD or ventriculostomy combined with antiplatelet therapy, DAPT, or tirofiban in aneurysm/SAC contexts; checklist, team, or communication in aneurysm neurointervention; and education, simulation, trainee, fellow, or junior in aneurysm neurointervention. The PubMed search was not restricted by language at the search-string level; for full-text interpretation and final citation selection, we used English-language original articles and major reviews. A screening workbook was used to consolidate duplicate records and classify each unique title/abstract as priority read, secondary read, or low directness for this case-based review. Detailed search blocks and first-pass screening counts are provided in Supplementary Material S1, and the cleaned screening log is provided in Supplementary Table S1. The search was used to prioritize articles that mapped directly onto the case timeline: pre-rupture risk sharing, early rupture recognition, primary hemostasis, the post-hemostasis thrombotic phase, transition to computed tomography (CT), external ventricular drainage (EVD), and intensive care unit (ICU) care, and team communication or education. The case description was prepared with reference to the CARE explanation and elaboration document, and the review structure was guided by the Scale for the Assessment of Narrative Review Articles (SANRA) [13,14]. Numerical data are therefore cited to support case interpretation; formal pooled estimates or risk-of-bias assessments are not presented.

## 3. Detailed Case Description

A 71-year-old woman developed dizziness and was diagnosed with coronavirus disease 2019 (COVID-19) at the initial presentation. Persistent dizziness and gait disturbance led to magnetic resonance imaging (MRI) at another hospital 6 days later, which showed a right cerebellar infarction and poor visualization of the basilar artery. She was referred to the treating hospital. On admission, she had left-sided weakness, left facial weakness, dysarthria, and ataxia. Edoxaban (30 mg) and argatroban were initially used, but follow-up MRI showed multiple new infarcts involving the cerebellum and brainstem. After transfer for rehabilitation approximately 1 month after symptom onset, she was readmitted 3 days later because of vomiting and left-sided weakness. Computed tomography angiography (CTA) performed during this readmission showed right vertebral artery occlusion, severe left vertebral artery stenosis, and the unruptured AComA aneurysm. Because recurrent ischemic events had occurred under anticoagulant-based treatment and severe vertebral stenosis remained, antithrombotic therapy was changed to aspirin 100 mg/day and prasugrel 3.75 mg/day as DAPT. Balloon angioplasty for the left vertebral artery stenosis was performed during the same readmission, and posterior circulation flow partially improved.

During the same readmission, the CTA-detected AComA aneurysm was reassessed. The aneurysm arose in a left A1-dominant A1-A2/AComA complex, projected posteriorly, and had a bleb. Right internal carotid artery (ICA) Matas testing did not show right A1 filling or cross-flow through the AComA. Preprocedural platelet-function testing was not performed before SAC. After rehabilitation, she could walk with a walker and eat independently; her modified Rankin Scale (mRS) score had improved to 2. The treatment decision was narrow: AComA location, left A1 dominance, wide neck, and a bleb supported concern for future rupture, particularly under continued DAPT, whereas the patient remained in the subacute period after multiple ischemic strokes, shortly after vertebral angioplasty, and soon after DAPT initiation. Delayed treatment after further stabilization would also have been reasonable. After discussion with the patient and family, SAC was selected during the same admission, approximately 2 months after the initial presentation.

**Figure 1:**
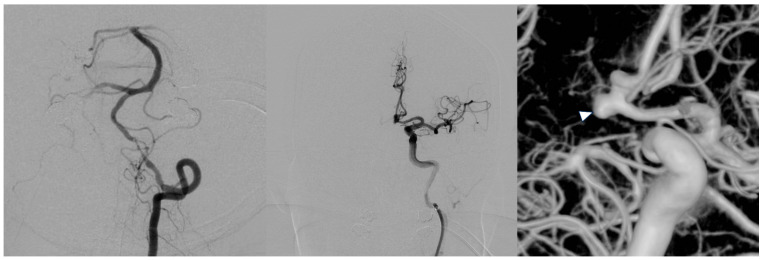
Preprocedural angiographic assessment of the anterior communicating artery (AComA) aneurysm. From left to right, left vertebral angiography after balloon angioplasty shows partial restoration of posterior circulation flow, left internal carotid angiography shows the AComA region in a left A1-dominant configuration, and three-dimensional rotational angiography shows a posteriorly directed AComA aneurysm with a bleb. The white arrowhead indicates the bleb.

A right femoral artery approach was used. A 6-Fr guiding system was placed in the high cervical segment of the left ICA. Before advancing the guiding system, left vertebral angiography confirmed residual stenosis but antegrade perfusion to both posterior cerebral arteries and superior cerebellar arteries. Heparin (5000 U) was administered intravenously. Activated clotting time (ACT) was maintained at 250–300 s with hourly monitoring. Intracranial digital subtraction angiography (DSA) and three-dimensional rotational angiography showed an aneurysm measuring 5.1 × 4.5 × 4.0 mm, with a 3.6-mm neck and a dome-to-neck ratio of 1.4 (Figure 1). A stent-delivery microcatheter (XT-17; Stryker Neurovascular, Fremont, CA, USA) was advanced into the distal right A2 segment, and a second microcatheter (SL-10; Stryker Neurovascular) was placed in the aneurysm. After insertion of an Axium Prime 3D 3.5 × 6 mm framing coil (Medtronic, Irvine, CA, USA), a Neuroform Atlas 3 × 15 mm stent (Stryker Neurovascular) was deployed to complete the jailed construct. Angiography after stent deployment did not show rupture or thrombus. Additional Axium Helix 3 × 4 mm and 2 × 4 mm coils were inserted with stepwise downsizing.

**Figure 2:**
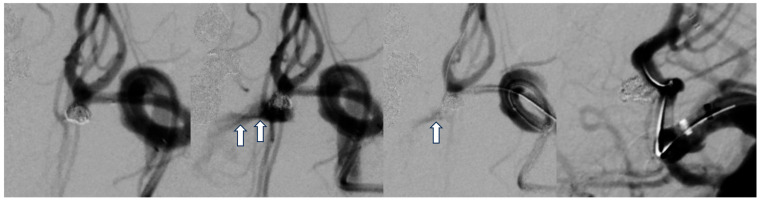
Hemorrhagic phase during stent-assisted coiling. Sequential working-angle angiograms obtained during insertion of the fourth coil show new contrast extravasation from the anterior communicating artery aneurysm. Temporary flow-control maneuvers reduced but did not immediately eliminate the leak. White arrows in the second image indicate the initial contrast extravasation at the rupture site, and the white arrow in the third image indicates slight residual extravasation after temporary flow-control maneuvers.

During insertion of the fourth coil (Axium Helix 2 × 3 mm), tactile resistance increased when approximately 5 mm of the coil remained. Immediate angiography showed new contrast extravasation, and the event was judged to be coil-related IPR (Figure 2). The time of angiographic rupture recognition was defined as T0. The anesthesia team was asked to lower systolic blood pressure below 120 mmHg, and heparin reversal with 5 mL of protamine was started. Five minutes later, ACT decreased from 278 to 137 s. A Shouryu 2 HR 3 × 5 mm microballoon (Kaneka Medix, Osaka, Japan) was advanced for neck control, but it could not pass the inlet of the deployed stent. The strategy was therefore changed to temporary occlusion of the right A1 segment, and flow was controlled for approximately 6 min beginning about 13 min after T0. Approximately 24 min after T0, slight residual extravasation remained. Sandwich closure through the residual jailed aneurysm microcatheter was attempted, but the SL-10 slipped into the stent and kicked back to the A1 segment, making re-navigation difficult.

**Figure 3:**
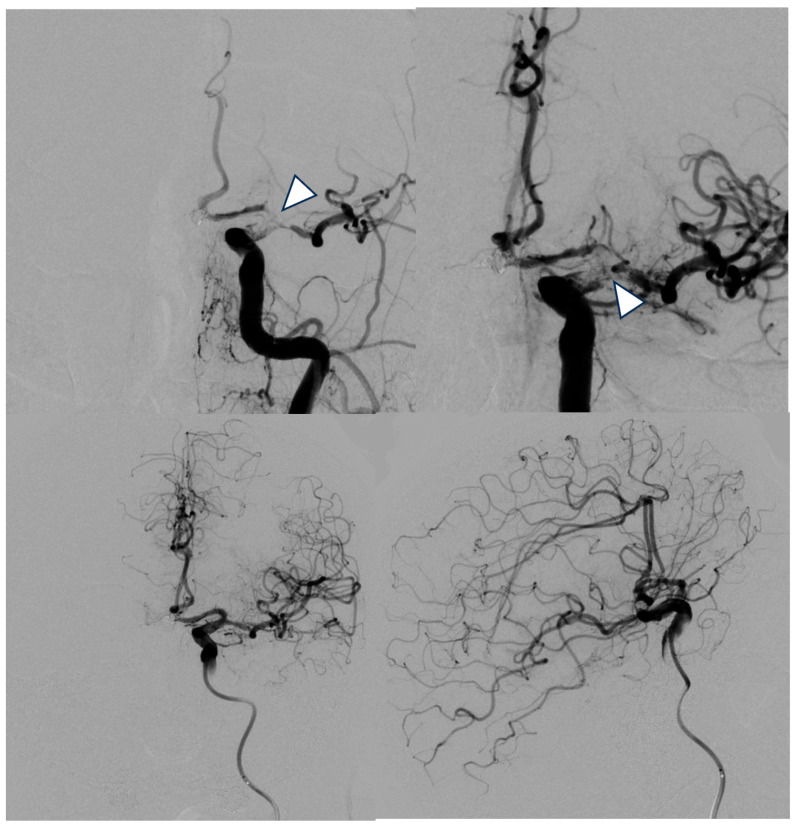
Thrombotic phase after hemostasis and angiographic recovery after rescue thrombectomy. Upper-row angiograms obtained after disappearance of extravasation show new filling defects involving the internal carotid terminus and the proximal A1 and M1 segments. Lower-row final angiograms after rescue thrombectomy show restoration of distal flow without recurrent extravasation. White arrowheads indicate filling defects.

After a trend toward hemostasis was confirmed, a global run was obtained. Approximately 40 min after T0, new filling defects appeared from the ICA terminus to the M1 and A1 segments (Figure 3). Heparin (3000 U) was administered 3 min later, and edaravone 30 mg was infused. ACT was 196 s approximately 53 min after T0. Rescue thrombectomy was performed using a Phenom 21 microcatheter (Medtronic) and an EmboTrap 5 × 35 mm stent retriever (Cerenovus, Galway, Ireland). Intra-arterial ozagrel (40 mg) was administered for mechanical spasm around the middle cerebral artery bifurcation. Final angiography approximately 73 min after T0 showed no recurrent extravasation and restoration of distal flow. The final ACT was 208 s, and the patient was returned to the intensive care unit (ICU) while intubated.

**Figure 4:**
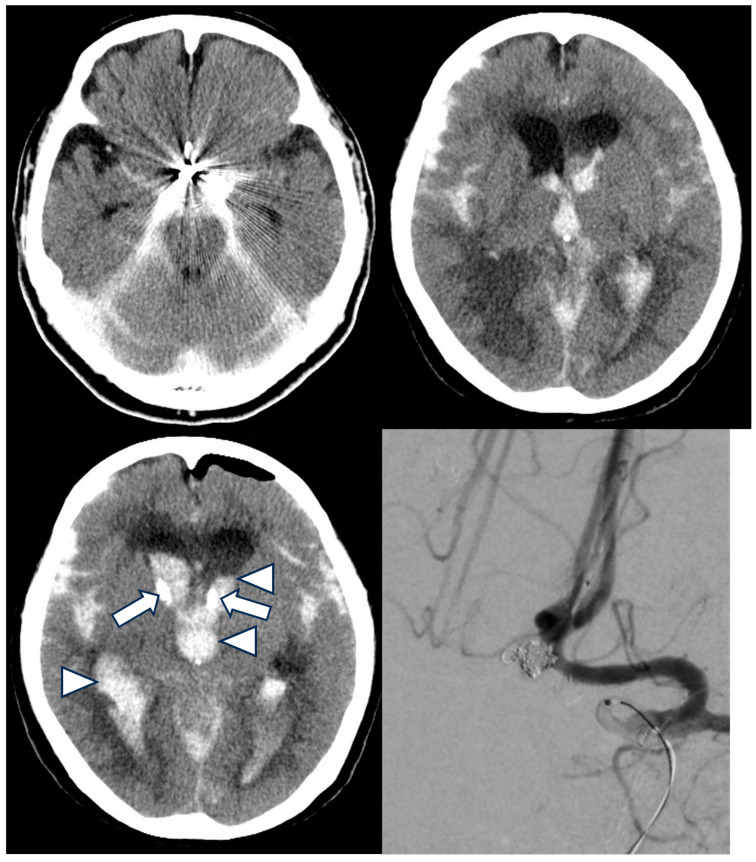
Postprocedural hemorrhagic progression and additional treatment. From upper left to lower right, immediate computed tomography (CT) shows basal cisternal subarachnoid hemorrhage (SAH) and intraventricular hemorrhage (IVH), follow-up CT after anisocoria shows progression of IVH with acute hydrocephalus, CT after bilateral external ventricular drainage (EVD) shows persistent IVH after emergency cerebrospinal fluid diversion, and next-day angiography shows additional trans-cell coiling. Because persistent aneurysmal bleeding could not be excluded on the basis of CT progression, additional trans-cell coiling was performed on the following day. White arrowheads indicate representative IVH, and white arrows indicate the bilateral EVD catheter trajectories/tips on the post-drainage CT image.

The complication sequence did not end in the angiography suite. Immediate CT obtained after return from angiography showed subarachnoid hemorrhage (SAH), mainly in the basal cisterns, with intraventricular hemorrhage (IVH) (Figure 4). Later the same day, anisocoria developed in the ICU. Repeat CT showed progression of basal cisternal and intraventricular hematoma with acute hydrocephalus. Bilateral EVD was performed early the following day, approximately 2 h after anisocoria was recognized, with both drains set at 10 cm H_2_O. Cerebrospinal fluid diversion was established, and anisocoria improved after EVD. Repeat angiography was then performed. No clear extravasation or thrombus was seen. However, persistent aneurysmal bleeding could not be excluded because increased intracranial pressure might have suppressed angiographic leakage and CT showed hemorrhagic progression around the basal cisterns and ventricles. Additional trans-cell coiling was therefore performed. The final angiogram showed no abnormal findings. The patient died on postoperative day 7, with an mRS score of 6.

## 4. Discussion

### 4.1. What This Case Adds to Neurointerventional Education

The educational value of this review lies in the compressed sequence rather than the rarity of IPR. In this patient, an AComA aneurysm with a bleb and a relatively small neck, a deployed intracranial stent, acute heparin reversal, temporary flow reduction, thrombotic occlusion, and later SAH/IVH with hydrocephalus occurred within one care pathway. Surveys and observational data indicate that technical execution, indication decisions, and unexpected-event management all contribute to complications, and that many elective coiling complications are detected during the procedure or within 6 h of anesthetic emergence [1,15]. IPR is uncommon but associated with worse outcomes [16]. For training, the practical question is how a junior team member can recognize the shift from bleeding to thrombosis and then to postprocedural hydrocephalus and communicate the next useful observation without delay.

### 4.2. Indication and Timing as a Narrow-Margin Decision

Before bailout management is considered, the first lesson from this case is decision-making. Rupture risk in UIA is not determined by size alone. UCAS Japan showed that natural history varies by size, location, and shape, and the PHASES score incorporates population, hypertension, age, aneurysm size, earlier SAH, and site [17,18]. The American Heart Association/American Stroke Association guideline recommends individualized decision-making based on aneurysm factors, patient factors, treatment risk, and patient preference [19]. In the present case, AComA location, a bleb, left A1 dominance, and a wide neck supported concern for future rupture. However, treating a 5-mm incidental UIA during the same admission as recurrent vertebrobasilar ischemia concentrated several risks: recent multiple ischemic strokes, recent vertebral angioplasty, newly initiated DAPT, general anesthesia, systemic heparinization, and the possibility that EVD would be required under antiplatelet exposure if IPR occurred. Delayed treatment after further stabilization of ischemic events and antithrombotic management was a reasonable alternative. Early SAC was selected after shared decision-making because posterior circulation flow had partially improved after angioplasty, functional status had improved to mRS 2, the patient and family preferred preventive treatment after explanation, and the long-term risk of the AComA morphology under continued DAPT was considered clinically meaningful. This case should therefore not be framed as an aneurysm treated simply because it was found; it was a narrow-margin decision in which rupture prevention and perioperative fragility were explicitly balanced. In this context, decision-making may be more consequential than a bailout maneuver once the complication has begun. The procedural briefing should include this decision map before the first catheter is inserted.

### 4.3. Pre-Rupture Risk Mapping

Preprocedural risk mapping should be more specific than saying, ‘this is a difficult AComA aneurysm.’ Aoki and colleagues identified bleb, small neck, and AComA location as independent risk factors for IPR during UIA coiling, and Kawabata and colleagues also reported small dome size and AComA as independent risk factors [7,20]. Schuette and colleagues found that in AComA aneurysms, those smaller than 4 mm had a higher IPR incidence than larger aneurysms [21]. AComA SAC series and meta-analytic data show that thromboembolism, procedural leakage, rupture, and coil prolapse can coexist as relevant procedural risks [9,10,11]. These data should be interpreted cautiously, because some AComA stent or anatomic studies include mixed ruptured and unruptured populations, and the study of IPR and thrombus formation in AComA coiling was based on ruptured aneurysms [10,22,23]. We therefore used these reports to map anatomical and complication signals, not to estimate the absolute risk for the present unruptured case. Stent-assisted embolization is a routine option for this anatomy, but the bailout map changes once the stent is deployed [24]. The practical map should include the bleb location, neck shape, A1 dominance, whether a neck balloon can realistically pass, where temporary flow control can be obtained, and how the jailed microcatheter route might be lost after stent deployment.

### 4.4. Early Recognition: The First Sentence Aligns the Room

Early recognition depends on converting a vague concern into a short statement that aligns the room. Ihn and colleagues noted that the first radiographic sign of perforation may be a device crossing the aneurysm boundary on the roadmap, followed by systemic changes such as blood pressure elevation or tachycardia [5]. In Aoki’s IPR series, most rupture sites were at the dome and the causative device was most often a coil [7]. In the present case, increased tactile resistance during insertion of the fourth coil followed immediately by contrast extravasation was a warning sequence consistent with coil-related IPR. Vital signs also matter: Kim and colleagues found that maximum systolic blood pressure after IPR and deviation of mean blood pressure from baseline were independently associated with poor outcomes [25]. Cho and colleagues reported that rapid occlusion of the suspected leakage point was performed in all IPR cases in a UIA series with generally favorable outcomes [26]. In the first seconds, the assistant does not need a long explanation. The useful prompt is brief and observable: ‘Resistance has changed,’ ‘Extravasation is suspected,’ and ‘Please confirm with minimal contrast.’ Those sentences trigger the sequence assumed by existing checklists: confirmation, blood pressure control, reconsideration of anticoagulation, preservation of tamponade, and continued packing when possible [3,4].

### 4.5. Primary Hemostasis and the Protamine Dilemma After Stent Deployment

Primary hemostasis during IPR requires several actions to run in parallel: blood pressure reduction, minimal contrast confirmation, continued coil packing, neck balloon or proximal flow control, and reconsideration of anticoagulation. The perforating coil or microcatheter itself may provide temporary tamponade and should not be withdrawn reflexively. Additional packing through the same or a second microcatheter, neck balloon protection, and proximal flow control remain key options [5]. Earlier reports by Hirai, Li, Tummala, and Levy also place continued packing and preservation of a hemostatic route at the center of perforation management [27,28,29,30]. Aoki and colleagues reported that, when a balloon guiding catheter was available, flow control could be achieved within seconds and hemostasis within minutes in many UIA-IPR cases [7]. Persistent contrast leakage beyond 10 min can still be controlled by additional coil packing or stenting in selected series, suggesting that the operative focus should remain on preserving a mechanical route rather than being governed by the clock alone [31]. Manual common carotid compression has also been reported as a proximal-control option when balloon access is not immediately available [32].

In this case, the most difficult moment was the decision to reverse heparin after Neuroform Atlas deployment. Protamine was given immediately after extravasation was recognized, and ACT fell from 278 to 137 s. A neck balloon could not cross the stent inlet, temporary right A1 flow control was used, and new filling defects appeared from the ICA terminus to the M1 and A1 segments shortly thereafter. Protamine cannot be assigned as the sole cause of thrombosis, because stent placement, temporary flow reduction, local coagulation after rupture, and ACT reduction all overlapped. Still, heparin reversal plausibly contributed to the thrombotic phase. In a multicenter registry, heparin reversal during IPR was independently associated with ischemic lesions [12]. A useful prompt at this point should not be a simple yes-or-no judgment about protamine. A safer formulation is to name the dilemma: ‘The stent is already deployed. Complete reversal may increase in-stent thrombosis. We need to confirm the hemostatic route and check the stent lumen and A1/M1 immediately after reversal.’ This puts drug choice, mechanical hemostasis, and thrombus surveillance onto the same timeline.

Another practical point is catheter-position awareness. During the attempted sandwich closure, the jailed SL-10 slipped into the stent and kicked back to the A1 segment. The lead operator did not immediately recognize the exact tip position. Not everyone in the room sees the same monitor or attends to the same detail during a crisis. A brief spoken update such as ‘The tip has returned to A1’ or ‘The microcatheter is now in the stent’ can protect the remaining hemostatic route. This is not a minor observation; it may determine whether additional packing, neck control, or proximal control remains possible. The same phase should also prompt explicit tracking of ACT, total heparin dose, protamine dose, stent deployment status, the viability of the jailed catheter, the feasibility of neck balloon passage, and the next angiographic run used to reassess stent patency and distal runoff.

### 4.6. The Second Phase After Hemostasis: Do Not Miss Acute Thrombotic Occlusion

The disappearance of extravasation can create a false endpoint [33]. In SAC-associated rupture, it may instead mark the start of a thrombotic phase. Nii and colleagues found acute in-stent thrombosis in 5.9% of UIA SAC procedures, with onset approximately 19 min after stent placement [8]. Senturk and Arat reported acute intraprocedural thromboembolism in 7.8% of endovascular procedures for UIAs and identified braided SAC and distal aneurysm treatment as risk factors [34]. Adeeb and colleagues reported postprocedural ischemic stroke and hemorrhage after treatment of intraprocedural thrombosis during SAC and flow diversion, underscoring that angiographic rescue does not end surveillance [35]. Thromboembolic complications after SAC of acutely ruptured aneurysms have also been associated with poor outcomes, and rescue strategies may require a combination of mechanical and pharmacological approaches [36,37]. In the present case, the filling defects appeared only after extravasation had improved. The immediate task is comparison rather than reassurance: each run should be compared with the previous one for the stent lumen, ICA terminus, A1, M1, and distal runoff. Prompts such as ‘The leak has stopped, but M1 is weaker than the previous run’ can shorten the time to rescue thrombectomy.

### 4.7. Beyond the Angiography Suite: CT, EVD, and ICU Handoff

This case also shows that the rescue pathway after IPR extends beyond the angiography suite. Kameda-Smith and colleagues reported that most complications after elective coiling were detected during the procedure or within 6 h of anesthetic emergence [15]. Therefore, once hemostasis is obtained, CT, ICU transfer, and EVD readiness should already be prepared. The issue is complicated in SAC because antiplatelet exposure increases concern about ventriculostomy-related bleeding. Hudson and colleagues reported that DAPT increased radiographic EVD-related hemorrhage in aneurysmal SAH but did not significantly increase symptomatic hemorrhage [38]. A meta-analysis by Cagnazzo and colleagues found higher major hemorrhage under DAPT but no clear difference according to whether EVD was placed before or after endovascular treatment [39]. Other series have described ventriculostomy under DAPT or antithrombotic exposure with variable radiographic bleeding but limited symptomatic deterioration [40,41,42,43]. These data do not make EVD risk-free; rather, they support planning the transfer with the bleeding risk already named.

During this transition, one person must bridge rooms while the lead operator completes hemostasis, thrombectomy, and final angiography. Circulating nurses, anesthesia staff, ICU staff, and the neurosurgical on-call team should receive the same key items: CT transfer readiness, EVD equipment, blood product and coagulation information, DAPT exposure, ACT trends, protamine dose, and whether rescue thrombectomy was performed. In the present case, anisocoria developed later the same day, and emergent EVD followed the diagnosis of acute hydrocephalus. A handoff that includes antithrombotic exposure, heparin reversal, thrombus rescue, and the possibility of rebleeding is therefore a safety action, not an administrative formality.

In a hybrid angiography/CT/operating-room environment, management might have been operationally faster but not conceptually different. Immediate CT without a long transfer could have confirmed IVH and hydrocephalus earlier, and EVD or surgical decompression could potentially have been prepared while angiographic hemostasis and stent-patency checks were ongoing. If persistent aneurysmal bleeding had been strongly suspected, operating-room access could also have lowered the threshold for surgical backup. However, the core sequence would remain the same: secure or preserve a hemostatic route, avoid blind withdrawal of tamponading devices, balance heparin reversal against stent thrombosis, confirm distal flow, and hand off the antithrombotic and rupture timeline. A hybrid suite reduces handoff distance; it does not remove the need for a preassigned team script.

### 4.8. Making Communication Models Usable: Psychological Safety

The literature and this case point to the same educational need: a young assistant or fellow should not have to invent language during a crisis. Ospel and colleagues emphasized standardization, simulation training, and shared bailout strategies, and Goyal and colleagues argued that complication avoidance must include learning from experienced operators’ errors [1,2]. Fargen and colleagues reported improved staff communication after implementation of a neurointerventional checklist [6]. Gross and colleagues framed near morbidity as highly instructive. Nawka and colleagues showed the value of modular simulation for difficult aneurysm procedures, and Nakamura and colleagues reported that video-based learning can help transmit tacit knowledge on coil embolization [44,45,46]. Short spoken scripts should therefore be regarded not as challenges to the lead operator, but as tools that lower the psychological burden of speaking up and make safety behavior reproducible.

Even a good script fails if the room does not permit it. Edmondson defined psychological safety as a shared belief that the team is safe for interpersonal risk taking, and Nembhard and Edmondson showed that leader inclusiveness and professional status influence psychological safety and improvement efforts in health-care teams [47,48]. In an angiography suite, seniority and professional roles can make it difficult for a junior member to say, ‘Protamine may increase thrombosis because the stent is already in.’ To reduce that barrier, preprocedural briefing should explicitly authorize any team member to speak immediately about extravasation, stent-flow reduction, ACT, or pupil changes. The operator should receive such statements with closed-loop communication, and complications should be reviewed in blame-free debriefing. Psychological safety is therefore not a soft add-on; it is a technical precondition for timely dissent during a neurointerventional crisis.

Table 1 translates the staged case-based review into spoken prompts. The point is not that a junior assistant must always make the correct diagnosis. The point is to know what to watch, whom to address, and which short sentence can keep the team on the next step.

## 5. Limitations

This case report and narrative review have several limitations. First, it is anchored in a single case and cannot prove the superiority of any management choice. Second, the patient died on postoperative day 7, but no autopsy or long-term angiographic follow-up was available; the exact mechanism of late hemorrhagic progression cannot be proven. Third, the reconstruction of shared decision-making, DAPT exposure, heparin reversal, and EVD timing is based on the medical record and cannot reproduce every conversation with the patient and family. Fourth, preprocedural platelet-function testing was not performed, so the contribution of individual antiplatelet responsiveness cannot be assessed. Fifth, the literature review was intentionally selective and limited to English-language articles for final citation selection. Although targeted PubMed search blocks were used, the objective was not to capture all IPR literature or conduct a formal risk-of-bias assessment. Sixth, some AComA SAC or anatomic studies include mixed ruptured and unruptured cohorts, and the IPR/thrombus AComA coiling study was based on ruptured aneurysms, so extrapolation to this case should be undertaken cautiously [10,22,23]. Data on thromboembolic complications and EVD under DAPT also include ruptured aneurysm cohorts and non-AComA lesions [36,37,38,39,40]. Finally, the proposed communication model and psychological-safety recommendations are educational hypotheses derived from retrospective case analysis and published literature; they have not been prospectively measured as team behaviors. The prompts in Table 1 should therefore be treated as educational scaffolding rather than as a prescriptive management algorithm. Nevertheless, the educational question is narrow and practical: during SAC-associated rupture of an AComA aneurysm, what should the junior team member see, say, and prepare next?

## 6. Conclusions

IPR during SAC should be understood as a time-dependent hemorrhagic–thrombotic escalation rather than a single technical mishap. In the present case, a narrow-margin decision for preventive SAC was followed by IPR after stent deployment, heparin reversal, acute thrombotic occlusion, same-day hemorrhagic progression, acute hydrocephalus, and death. For trainees and fellows, angiographic hemostasis should not be regarded as the end of the crisis. It is the point at which surveillance for thrombosis, CT transfer, EVD readiness, and ICU handoff begins. The assisting role is to share the pre-rupture risk map, name early rupture, protect mechanical hemostatic routes, state the hemostasis–thrombosis dilemma after stent deployment, compare flow after hemostasis, and bridge the patient to the next care area. A stage-based, psychologically safe communication model may help convert rare catastrophic events into more reproducible team responses.

## Figures and Tables

**Table 1:** Time-dependent assistant-operator communication model for hemorrhagic–thrombotic escalation after intraprocedural rupture during stent-assisted coiling.

Phase	Cognitive Focus	Example Spoken Prompt	Main Addressee
Before rupture	Risk map and bailout route	This is a high-risk AComA aneurysm. Let us confirm where flow control can be obtained.	Lead operator
Suspected rupture	Confirm leak with minimal contrast	Resistance has changed. Extravasation is suspected. Please confirm with minimal contrast.	Lead operator/radiology technologist
Primary hemostasis	Preserve remaining hemostatic routes	Please do not withdraw that microcatheter yet. It may still be the tamponade route.	Lead operator/nurse
After stent deployment	Name the hemostasis-thrombosis tradeoff	The stent is already deployed. Reversal may increase thrombosis. We should check the stent lumen and A1/M1 immediately.	Lead operator/anesthesiologist
After hemostasis	Compare flow with the previous run	The extravasation has stopped, but M1/A1 looks worse than the previous run.	Lead operator/radiology technologist
Transition out of the angiography suite	Bridge to CT, EVD, and ICU	The operator is completing the final angiogram. Please prepare CT, EVD, and ICU handoff, including antithrombotic exposure.	Anesthesiologist/nurse/ICU and neurosurgical team

AComA, anterior communicating artery; CT, computed tomography; EVD, external ventricular drainage; ICU, intensive care unit.

## Data Availability

The data underlying this article cannot be shared publicly because they contain confidential clinical information from an individual patient. Relevant anonymized data may be provided by the corresponding author upon reasonable request, subject to institutional and ethical restrictions.

## Supplementary Materials

The following supporting information can be downloaded at https://www.mdpi.com/article/10.3390/jcm15114056/s1, Supplementary Material S1: Literature search and first-pass screening summary. Supplementary Table S1: Cleaned screening log for the targeted case-based narrative review.
