# Supplementary material for: Case Report: Hemorrhagic–Thrombotic Escalation After Intraprocedural Rupture During Stent-Assisted Coiling: A Case-Based Narrative Review and Staged Communication Model
Source: J Clin Med. 2026 May 24;15(11):4056. doi: 10.3390/jcm15114056 (PMC13257687; doi:10.3390/jcm15114056)
Supplement: Supplementary file 1 [file jcm-15-04056-s001.zip › Supplementary Material S1.pdf]

Supplementary Material S1

Literature search and first-pass screening summary

For: Case Report: Hemorrhagic-Thrombotic Escalation after Intraprocedural Rupture during Stent-Assisted Coiling: A Case-Based Narrative Review and Staged Communication Model

Purpose and scope

This supplementary material documents the targeted literature-search support used for a Case Report with a case-based narrative review. The search identified original studies and major reviews that mapped onto the time-dependent sequence emphasized in the clinical case: pre-rupture risk mapping, early rupture recognition, primary hemostasis, the post-hemostasis thrombotic phase, transition to computed tomography, external ventricular drainage, and intensive care, and team communication or education. It was not designed as a systematic review or scoping review, and no formal pooled estimate or risk-of-bias assessment was planned.

Database, date, and search structure

Database: PubMed. Search date: 31 March 2026. Fields: title/abstract text-word blocks. The seven concept blocks intentionally overlapped so that papers relevant to more than one phase, such as rupture plus hydrocephalus or stent-assisted coiling plus thrombosis, could be captured and later consolidated. The strategy is provided to document the scope of a targeted narrative search for a case-based review, not to support PRISMA flow reporting or exhaustive evidence synthesis. The full PubMed title/abstract syntax is provided in the companion screening workbook, Supplementary Table S1. The PubMed search was not restricted by language at the search-string level; however, the final narrative synthesis and citation selection were limited to English-language original articles and major reviews.

Seven PubMed concept blocks and first-pass counts

Block	Concept	Read now	Read secondary	Excluded	Total memberships
Q1	IPR / aneurysm perforation during coiling or SAC	28	26	360	414
Q2	AComA aneurysm with rupture, perforation, extravasation, or endovascular-treatment context	21	50	131	202
Q3	Acute thrombotic or thromboembolic events after SAC	19	25	86	130
Q4	Rerupture, IVH, or hydrocephalus after IPR or aneurysm perforation	8	8	8	24
Q5	EVD or ventriculostomy under antiplatelet exposure in aneurysm/SAC contexts	23	14	27	64
Q6	Checklist, team, or communication in aneurysm neurointervention	4	4	32	40
Q7	Education, simulation, trainee, fellow, or junior in aneurysm neurointervention	6	21	23	50

Note. Counts are block memberships before cross-query consolidation. Because records could appear in more than one block, the block-membership total equals the raw hit count and exceeds the number of unique records.

Record flow and screening categories

Item	Count
Raw records across seven PubMed block exports	924
Duplicate occurrences across query blocks	70
Unique records after DOI/title consolidation	854
Priority records for immediate full-text assessment or detailed citation mapping (Read_now)	88
Secondary records retained for possible background use (Read_secondary)	130
Records excluded for low directness to this focused case-based review	636
References ultimately cited in the submitted manuscript	48

Screening-category definitions

Screening category	Definition used for this review
Read_now	Papers directly usable in the manuscript: intraprocedural rupture recognition or hemostasis; AComA-specific risk and SAC context; acute thrombotic phase after SAC; rerupture, hydrocephalus, or EVD under antiplatelet exposure; or checklist, communication, simulation, and education relevant to assistant-focused teaching.
Read_secondary	Supportive papers that might strengthen background or discussion but were not required for the first manuscript pass, including broader AComA series, small/tiny aneurysm literature, broader thromboembolic or antiplatelet papers, and team/education background papers.
Exclude	Papers with low directness to this focused question. This category does not indicate low quality; common reasons were non-intracranial or non-neurovascular embolization, letters/replies/editorials without usable data, or general aneurysm papers not directly related to the assistant-focused case-based review.

Thematic distribution of retained records

Theme	Read now	Read secondary	Total retained
AComA lesion-specific context	14	35	49
SAC acute thrombosis / antiplatelet	13	30	43
Intraprocedural rupture / hemostasis	32	10	42
Hydrocephalus / EVD / hemorrhage	20	5	25
Education / team	0	24	24
Small / tiny aneurysm context	0	16	16
General complication background	0	10	10
Education / simulation	5	0	5
Checklist / team communication	4	0	4

Use of the screening set in the manuscript

The final reference list was not generated by mechanical inclusion of all Read_now records. Instead, the screening set was used to map the literature to the manuscript's time-dependent educational structure. Original studies and major reviews were prioritized when they directly supported one of the following manuscript components: AComA-specific risk and SAC context; IPR recognition and hemostasis; the

protamine/hemostasis-thrombosis dilemma; acute thrombosis after SAC; EVD and postprocedural transition under antiplatelet exposure; and neurointerventional checklist, simulation, or communication training.

Abbreviations

AComA, anterior communicating artery; DAPT, dual antiplatelet therapy; EVD, external ventricular drainage; ICU, intensive care unit; IPR, intraprocedural rupture; IVH, intraventricular hemorrhage; SAC, stent-assisted coiling.
